# A multi-scale investigation of the human communication system's response to visual disruption

**DOI:** 10.1098/rsos.211489

**Published:** 2022-04-13

**Authors:** James P. Trujillo, Stephen C. Levinson, Judith Holler

**Affiliations:** ^1^ Donders Institute for Brain, Cognition and Behaviour, Radboud University Nijmegen, The Netherlands; ^2^ Max Planck Institute for Psycholinguistics, Wundtlaan 1, 6525XD Nijmegen, The Netherlands

**Keywords:** kinematics, gesture, speech, compensation, multimodal

## Abstract

In human communication, when the speech is disrupted, the visual channel (e.g. manual gestures) can compensate to ensure successful communication. Whether speech also compensates when the visual channel is disrupted is an open question, and one that significantly bears on the status of the gestural modality. We test whether gesture and speech are dynamically co-adapted to meet communicative needs. To this end, we parametrically reduce visibility during casual conversational interaction and measure the effects on speakers' communicative behaviour using motion tracking and manual annotation for kinematic and acoustic analyses. We found that visual signalling effort was flexibly adapted in response to a decrease in visual quality (especially motion energy, gesture rate, size, velocity and hold-time). Interestingly, speech was also affected: speech intensity increased in response to reduced visual quality (particularly in speech-gesture utterances, but independently of kinematics). Our findings highlight that multi-modal communicative behaviours are flexibly adapted at multiple scales of measurement and question the notion that gesture plays an inferior role to speech.

## 1. Introduction

During everyday face-to-face interaction, we are frequently confronted with environmental challenges in communicating. This may be due to background noise, such as other people speaking, or reductions in visual quality due to occlusions or poor lighting. Despite these potential difficulties, people are typically able to communicate effectively. Investigating how we adapt our communicative strategies to such challenging situations will advance our understanding of how dynamic and flexible human communication is. The present study contributes to this endeavour by focusing on how the human communication system responds to the disruption, or decreasing availability, of visual communicative signals.

In terms of studying signal adaptation to disruption, several studies have focused on disruption of the *auditory* channel, and the so-called Lombard effect that it produces. The Lombard effect describes the way speakers modulate the acoustics of their speech, such as intensity and pitch, in response to auditory noise [1,2]. By incorporating the visual channel, it has also been found that speakers additionally modulate their lip movements in response to auditory noise [3,4]. More specifically, when their addressee can see them, speakers reduce the auditory (i.e. speech acoustic) modulation while increasing the visual (i.e. lip kinematic) modulation [5,6]. These studies have contributed to our understanding of how speech is adapted to the communicative context, and they point towards visual speech (lip and mouth movements) being able to compensate when the auditory signal is disrupted.

Building on the studies of audio-visual speech described above, a recent study showed that a *multi-modal Lombard effect* also extends to co-speech-gesture kinematics. Hand movements produced together with speech (co-speech gestures) play a prominent role in communication and are considered to form an integral part of human language [7–10]. In fact, speech and co-speech gestures are thought to arise from one common conceptual planner during utterance production [11–14] .Trujillo *et al*. [15] found that the kinematic form of these gestures is adapted in response to disruptive auditory noise [15], thus suggesting that communicative adaptation to auditory noise is in fact a multi-modal, embodied phenomenon. Similar to the studies of audio-visual Lombard effects, this study also found that acoustic adaptation was reduced when the speech was paired with co-speech gestures [15]. These studies show that both the auditory and visual channel are adapted in response to auditory disruption, and this seems to be an integrated response, with auditory adaptation being less pronounced when paired with visual signals.

The studies mentioned above underline the inherently multi-modal nature of human communication and have provided important insights into how the auditory and visual channels work together to achieve communicative success when auditory communication is not fully effective. However, in the context of discussing how the human communication system responds to environmental challenges, the focus on auditory disruption could bias towards assuming the visual channel is secondary to the auditory channel, and primarily serves to support or compensate a compromised auditory signal. For instance, accounts of language evolution and acquisition often frame gesture as fulfilling a sort of ‘bridging function’. In this framing, gesture primarily helps communication when spoken language does not yet provide a fully fledged communication system, with speech eventually replacing gesture as the primary carrier of information [16–20]. Similarly, the visual channel has been argued by some to be primarily speech-facilitating in nature, such as in the context of lexical retrieval (e.g. [21,22]) rather than communicative in its own right. However, others have argued that auditory and visual signals are tightly integrated, forming one multi-modal system in which the two modalities share equal (though often complementary) roles in the communication process [8,10,23–27]. Further elucidating the role of gesture in human communication is necessary in order to more fully understand the nature of the human communication system and the core question of how adaptable to environmental constraints it is.

Using a reverse Lombard paradigm in which the visual channel, rather than the auditory channel, is disrupted would thus allow us to better assess the status of gesture in mastering challenging communication. Specifically, such a paradigm would allow us to ask whether the auditory channel also compensates for visual loss, which would be informative about the importance of the visual channel relative to the auditory channel, as well as the interaction of visual signals with speech.

In addition to the question of whether communicative adaptability is a two-way street (with speech also adapting to gesture, not just the other way around), an interesting question is to what extent gesture itself adapts when gesture visibility is compromised. Previous research has manipulated visibility between interactants and shown that speakers more or less cease to gesture, at least when they talk in monologue [23,28,29]. If they cannot see each other but continue to dialogically interact, such as on the telephone, then speakers continue to gesture to a considerable extent, and much more so than when speaking in a monologue while their addressee cannot see them, but significantly less so than during face-to-face dialogue [23]. This has been interpreted as the dialogic interaction with the other person triggering a social response that is closely tied to gesture, despite the gestures having no obvious communicative effect in such contexts. These are very intriguing findings, and they certainly support the idea that co-speech gestures do not just facilitate speech since they respond to manipulations of the social context.

However, what such studies cannot tell us is whether the gestural response to visibility disruption is a coarse-grained response, where as soon as speakers realize that their gestures cannot be seen they cease to produce them. In case speakers are still engaged in dialogue and continue to gesture (albeit less than when visible), this, too, may be the result of a quite generic social response. This leaves open the question as to whether speakers would adapt both the relative frequency and form of their gestures in a targeted manner when visibility is not manipulated as an ‘all or nothing variable’, but by decreasing the visibility of gesture in a graded fashion. If speakers were to finely attune their gestural production to how much of their gestures can still be seen, this would be strong evidence that speakers adapt their co-speech gestures in a manner that is designed to overcome specific environmental challenges to communication that are *not* tied to challenged verbal communication. That is, such findings would point towards gesture being adapted to maximize gestures' communicative effect while taking into account how well they can or cannot be seen. This would suggest a fundamentally different mechanism than gestural adjustments resulting from a compensatory process governed by the verbal modality being compromised.

### 1.1. Current study

To summarize, we are here interested in whether (i) speech also adapts to support gesture, especially in a context in which visibility is compromised but the verbal channel is not inflicted with any disruptions; (ii) whether gesture itself adapts to environmental challenges of communication when these challenges are not associated with compromised verbal communication, specifically we are asking whether gesture is adapted in a fine-grained, carefully controlled manner that targets the specific visibility conditions during communication; and (iii) whether/how speech and gesture dynamically interact in the process of adapting to visibility reduction. In order to address these questions, we used a reverse, parametric Lombard paradigm by manipulating the quality of the video channel during video-mediated conversational interaction, providing 10 steps of decreasing visual quality. This approach allowed us to assess how the auditory and visual channels work together, and whether communicative adaptations scale with the degree of disruption. In other words, signal adaptation should increase with increasing disruption, but only to the point that adaptation is likely to be communicatively effective.

Regarding speech, we may observe no acoustic adaptation, which would be suggestive of compensatory processes flowing one way, from gesture to speech, but not the other way around. Such a finding may be in line with a notion of speech inhabiting a special status in human communication, which in some ways is superior to gesture. Alternatively, we may observe that speech does acoustically adapt to a decrease in visibility as an attempt to increase communicative success, even if this may not necessarily be effective, similar to gestures being produced on the telephone. Such a finding would put the speech and gesture modalities on a more equal footing. Additionally, if speech adapts to decreased visibility, we expect speech acoustic adaptation to follow either the same (i.e. coarse-grained or graded), parallel response to gestures, or in a complementary fashion (with effort invested into the verbal modality because the gesture is ineffective due to poor visibility).

Regarding gestures, we may observe a rather suddenly reduced relative frequency of gesture and kinematic salience at a certain level of visual degradation when gestures are deemed to lose their communicative effectiveness, without any evidence of graded adaptation. This outcome would be indicative of a rather coarse-grained form of social adaptation of the gesture signal, with gestures being deemed as useful when decipherable but simply dropped (rather than adapted) when this is no longer so. Alternatively, gestures may be gradually adapted (i.e. gradually increased in rate and kinematic salience) in response to visual quality reduction, up to the point where visual quality is so low that any adaption would no longer sustain effective gestural communication. This would suggest that the adaptation process is linked to a detailed awareness of the environmental conditions and how changes in gestural form can target these conditions to be as communicatively effective as possible.

**We take a multi-scale (three-level) approach to assess acoustic and gestural adaptation**, using variables that have been linked to signal salience and communicative effectiveness in past research [2,15,30–35]. Specifically, we hypothesize that evidence for adaptation will be present in the communicative signals at multiple scales of investigation. At the **articulator level,** we look at how manual movement fits together with movement of multiple other bodily articulators. For this analysis, we calculate *motion energy* of individual visual articulators (i.e. head, torso, arms and fingers) in order to determine whether visual signals are only used when they are visible (e.g. finger motion being reduced when the fingers are less clear, while torso motion remains until the speaker is essentially no longer visible), or whether the visual articulators follow a more general pattern of adaptation with all articulators increasing and decreasing/levelling out at approximately the same visibility conditions. This will provide insight into how communicative effort is distributed during adaptation of the visual channel and how fine-grained a process the adaptation is. Zooming in to gesture as a system together with speech, we investigate the **system level**. Here, we expect adaptation to manifest as a tighter coupling between speech and gesture, as previous research has shown that speech-gesture coupling (i.e. the time delay between acoustic peaks and kinetic peaks) is also sensitive to communicative context [36], and this tight coupling may support speech-gesture integration [37], which may in turn facilitate comprehension for the addressee. We therefore calculate the *coherence* (i.e. strength of coupling) as well as phase *asynchrony* (i.e. temporal offset between speech and gesture). Finally, we further zoom in to our main signals of interest (i.e. speech and gesture) at the **signal level**. Here, we investigate adaptation at the level of gesture kinematics and speech acoustics. In contrast with the articulator level, which focused on a general quantification of movement, measurements at this level will quantify the characteristics of the signals being produced. For gesture, we quantify the effect in terms of spatial and temporal kinematic features which have previously been linked to communicative adaptation [15,30]. Specifically, *hold-time* characterizes the temporal segregation of a gesture, which can make it easier to understand the movement [38], *peak velocity* captures the temporal salience of a movement and has been linked to communicative relevance [30], and *submovements* characterize the number of movements (i.e. potentially informative units) that make up a gesture and has shown to be increased when communicating in a noisy environment [15]. Peak velocity can be seen as a marker of salience at the level of individual movements, while submovements and hold-time represent how temporally segmented the gesture is, which may also indicate higher salience and interpretability of the gesture overall. *Depth* captures whether people extend their gestures towards their recipient, potentially as a way to reduce the perceived distance between speakers [39] in order to compensate for decreasing visibility. *Size* and *McNeillian space* capture spatial adaptation in terms of overall size (volume) and in terms of spatial prominence/salience (McNeillian space), as size and spatial prominence have been linked to communicative adaptation in child-directed interaction [40], in response to feedback [41], and when communicating information known to be relevant to the addressee [30]. Finally, *temporal variability* captures the rhythmic stability of the gesture, as greater rhythmicity may relate to communicative effectiveness [42,43]. For speech, we calculate *speech intensity* (loudness) and *pitch* (fundamental frequency), which have been linked to communicative adaptation in the original, auditory Lombard paradigms [2,31]. We expect that these features, as aspects of signal salience, will increase in response to decreasing visibility.

In sum, this study will determine how reduced visual quality during interaction affects the production and alignment of the visual auditory channels of multi-modal communication. This will provide insights into the flexibility of speech-gesture dynamics in adapting to environmental communicative demands.

## 2. Methods

### 2.1. Participants

Data were initially collected from 38 dyads (76 participants), recruited from the participant database of the Max Planck Institute for Psycholinguistics. Dyad recruitment involved requesting each participant who signed up to bring a friend with them to the experiment. Dyads were excluded if there were technical errors in the audio or visual recordings of either participant, leaving 20 dyads (40 participants total; 31 female, mean age: 24.23 years) for analysis. Participants were all native speakers of Dutch and were familiar with the other person in the dyad.

### 2.2. Apparatus and procedure

Dyads engaged in casual, unscripted conversation, mediated by a video-based telecommunication interface (similar to communicating via Skype or Zoom), for 40 min each. Dyads were informed that there was no ‘task’, but that they should simply engage in conversation for 40 min. Participants were only asked to avoid topics that would make their partner feel uncomfortable or embarrassed, and to try to ignore the experimental equipment and behave as naturally as possible. Participants in each dyad were located in separate rooms and saw and heard each other via a live audio-video feed. The video feed was displayed on a large screen (27″, 16 : 9, 1920 × 1080 px, full HD) directly in front of them and was generated in the following way: a specially built boxed apparatus filmed each participant from a frontal view. This video stream was fed into a computer where a custom-made script applied the blur manipulation core to the present paradigm (see below for details). Audio was recorded via small clip-on, highly sensitive microphones (Sennheiser EW 100 G3) that were attached to the box apparatus. The audio recording was fed onto the audio track of a separate video camera filming each participant from the side. The custom-made computer script also issued auditory beeps at a constant interval of 240 s (coinciding with the changes in blur grade, see below) which was fed into the same camera as the speech audio. A separate device (Atomos Ninja 2) recorded the manipulated video together with these two audio tracks. The audio beeps were inaudible to the participants and simply served the synchronization of the visual and audio streams from all recording devices (i.e. three video recordings [frontal camera, side camera, Ninja recorded-blurred-frontal video]) and one speech audio recording per participant). The synchronization was done manually (Adobe Premier Pro CS6), which served to overcome the 130 ms delay introduced by the process of blurring the video (note that this delay was a constant and concerned all interactions, also those in the no-blur condition since the technical set-up was the same, meaning the delay cannot account for condition differences). The manipulated video stream was then fed onto screens placed at the top of the boxed apparatus, facing down. Based on a one-way mirror construction inside the box, participants saw this visually manipulated video (i.e. as a frontal display of their conversational partner) rather than the video camera that filmed them. The size of the screen ensured that the displayed image was close to life-sized. Participants could hear their partner's speech via in-ear headphones, the cables of which were led over the shoulders and down the backrest to avoid interference with arm or hand movements.

**Figure 1.**
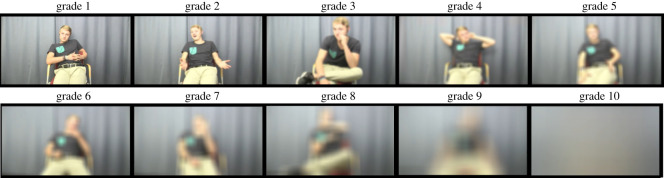
Visual depiction of the actual blur grades used in this study.

The custom-made computer script induced a change in visual quality in 10 separate steps of full-screen Gaussian blur, with each step occurring after 4 min (figure 1). The direction of quality change (clear to blur or blur to clear) was randomized across participants. Ten dyads went from blurry to clear, and 10 dyads went from clear to blurry. Conversations were not constrained, and participants were instructed to simply carry out a natural conversation for the duration of the experiment. The frontal and side video recordings were used in subsequent analyses (i.e. processed in OpenPose and used for gesture annotation, as described below). Participants also conversed for 4 min on the telephone and 4 min face-to-face (in counterbalanced order), but these data do not form part of the present analyses.

#### 2.2.1. Gesture annotation

**Figure 2.**
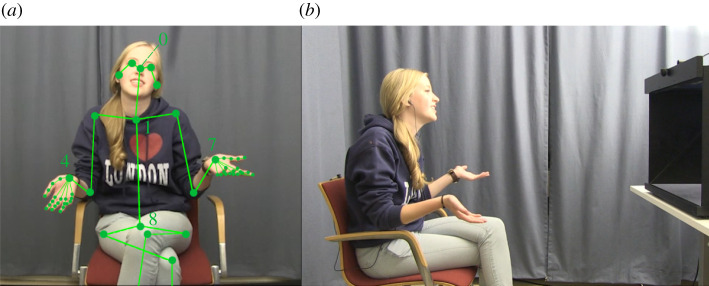
(*a*) Overview of set-up and tracked keypoints overlaid on a video still frame; (*a*) depicts the front-view as seen by the other participant. In this panel, the green circles additionally illustrate the tracked keypoints (not visible during the experiment), while lines are drawn between these keypoints to schematically depict the overall ‘skeleton’ of the tracked person. Note that only the keypoints themselves are used for analysis; (*b*) depicts the side-view (used for calculating kinematic depth parameter), to give an overview of the experimental set-up.

Before running analyses, we annotated all manual gestures in the data. This was done using the SPUDNIG application [44] which processes the videos in OpenPose [45]. OpenPose is an offline, video-based motion tracking algorithm that provides *x*,*y* coordinates of 25 keypoints across the body, including for example the shoulders, elbows and wrists (figure 2 for a schematic overview of keypoints). Keypoints are automatically detected in each frame of the video using a computer vision algorithm, providing frame-by-frame coordinate data, provided in pixels, for each of the keypoints. SPUDNIG uses these OpenPose data to detect all movements produced by each participant. These movements were then manually checked and non-gesture movements were removed. Reliability for gesture identification was based on 10% of the data (four minutes per participant). This subset of the data contained 12.7% of all gestures. A second coder manually annotated these data, and agreement reached 81%. After the removal of non-gesture movements, we annotated each gesture as being representational (e.g. iconics, metaphorics, McNeill, [9]), abstract deictics, [28], pragmatic (Kendon [8]; here defined as, e.g. beats, emphatics, mood and stance modifiers), emblem (e.g. ‘thumbs-up’, ‘cheering’, Ekman & Friesen, [46]) or interactive (e.g. palm-up open-hand, addressee-directed deictics, [47–49]). This was done to account for potential differences in kinematics and alignment across gesture types. In total we annotated 3587 unique gestures (*Representational*: *n* = 980, mean duration = 2836 ms, *Pragmatic*: *n* = 627, mean duration = 1728 ms, *Interactive*: *n* = 1897, mean duration = 1718 ms, *Emblem*: *n* = 83, mean duration = 2164 ms). Reliability of gesture categorization was substantial (Cohen's kappa = 0.75). See electronic supplementary material for more details on the gesture features, as well as reliability and coding criteria.

### 2.3. Analysis—features

#### 2.3.1. Articulator level

##### 2.3.1.1. Motion energy

In order to capture the amount of effort being put into the different articulators, we used an approach inspired by motion energy analyses. By using the keypoints captured by OpenPose motion tracking (figure 2*a*), we calculate how much movement is occurring within specific parts of the body while allowing us to ignore or account for movement in other parts of the body. Specifically, motion energy is operationalized as the sum of pixel change for a given keypoint (see below). In each frame of the motion tracking video, the change in position of a keypoint relative to the previous frame can be measured by the difference in *x*,*y* values (given in pixels) between the two frames. Taking the sum of these differences through the duration of a gesture event provides a quantification of the motion energy of a given articulator during this event. This is taken as a measure of how much effort and perhaps the amount of visual information (in the form of kinematic motion) that is produced in specific bodily articulators.

Using this approach, we calculated the motion energy of the *Torso,* the *Head*, correcting for torso motion, the *Arms,* correcting for torso motion, and the *Fingers*, correcting for arm motion. For the Head, we used the *nose* keypoint (keypoint 0). For the Torso, we used the keypoint for the upper body (keypoint 1); for the Arms, we used the keypoints for the hands (keypoints 4 and 7), as these are taken to capture the gross movements of the arms. For the fingers, we took the average energy for each of the five finger tips on each hand. For Arms and Fingers, left and right values were averaged.

#### 2.3.2. System level

##### 2.3.2.1. Speech-gesture alignment

Data were prepared for the speech-gesture alignment analyses following the methods described by Pouw, Trujillo & Dixon [50], synchronizing and merging the continuous kinematic data of the hands (i.e. the frame-by-frame velocity of the hand) together with the speech amplitude envelope, calculated with the Python package Parselmouth [51].

We then calculated speech-gesture alignment using cross-wavelet analysis (CWA), implemented in the R package ‘WaveletComp’ [52]. CWA effectively decomposes two time-series into discrete wavelets, allowing one to calculate the coherence, or correlation, between the two time-series, as well as other features of interest such as the phase asynchrony, which describes the temporal lag between components of the signal, such as the peaks. We proceeded by calculating coherence between speech and gesture between 2 and 8 Hz, meaning that the rhythmic structure of the signals was assumed to recur on a timescale between 125 and 500 ms. This effectively kept our window of analysis at syllable-length frequencies, ensuring that alignment could be calculated for even temporally short gestures. For each speech-gesture segment, coherence was tested for statistical reliability (*p* < 0.05). We extracted all statistically reliable **Coherence** (i.e. strength of coupling) and corresponding **Phase Asynchrony** (i.e. temporal offset) values in order to test for an association between visual quality and the CWA measures.

#### 2.3.3. Signal level

##### 2.3.3.1. Speech

In order to capture speech acoustics, we used the Praat software (Boersma & Weenink, [53]), implemented in Python via Parselmouth [51], to extract the **maximum intensity** (i.e. loudness) in decibels (dB) and the **maximum F0**. These values were selected due to their involvement in other communicative modulation, namely adaptation to auditory noise [6,31]. We used the gesture annotations (described above) in order to split the raw acoustic data into two time-series: one containing all speech produced together with gesture, and one containing all of the speech produced without co-occurring gesture. We then calculated the mean of each time-series, per blur grade, which provided one measure for speech-only acoustics, and one measure of speech-gesture acoustics.

##### 2.3.3.2. Gesture

**Gesture rate** was taken as the number of gestures per minute (i.e. gesture frequency divided by time in minutes, which is highly correlated with measuring rate by number of words, [23]) to account for differences in the amount of speech produced. Finally, we calculated kinematic features to address the question of how gestures are affected by decreasing visual quality.

Kinematic features were calculated based on the code provided in Trujillo *et al*. [54], using motion tracking data from keypoints 4 and 7. In short, we calculated **peak velocity**, defined as the maximum velocity value during a gesture, number of **submovements**, defined as the number of individual ballistic movements (e.g. strokes, repetition of strokes and preparatory movements), **hold-time**, defined as the amount of time during gesture executing (i.e. excluding rest before initial movement and after final movement) where the hands are still, gesture **size**, which is defined as the maximum extension of the hands into the *x* and *y* directions with respect to the body (i.e. volumetric size), and **McNeillian space**, which is based on McNeill's [10] delineation of gesture space into centre-centre, centre, periphery and extra-periphery, **Depth**, which captures how far away from the body the hands extended (defined as the distance between the hands and the hips (keypoint 8), using motion tracking data from a side-view camera (figure 2*b*), in order to capture changes due to simply moving the hands forward, but also due to moving the entire upper torso forward), and the normalized pairwise variability (nPVI; **Temporal Variability**), which quantifies the temporal variability of the gesture submovements [55,56]. Note that all features were calculated using motion tracking of a front-view camera (figure 2), except for depth, which used a side-view camera. Front-view camera was used for most features as we believe this captures the spatio-temporal kinematics that are most visible to the addressee. These features were selected due to their role in communicative signalling. In order to ensure that there was no multi-collinearity between the kinematic features, we calculated the variation inflation factor across features [57]. No values exceeded the recommended threshold of 3 [57], indicating that no features were colinear.

### 2.4. Analysis—statistical testing

All statistical tests were performed in R [58] using the lme4 package (Bates *et al*. [59]), implementing polynomial mixed effects regression analyses. Based on our *a priori* hypothesis regarding signals being adapted primarily in the initial blur grades but less so in later blur grades, our models contained a second-order polynomial of the blur grade as the primary independent variable. Separate tests were used for the features of interest, reiterated below, with each as the dependent variable in its own model. We built up the random effects structure of the model by first testing which structure was the best fit to the data using chi-square tests. We first included dyad and participant as nested random effects, then added gesture type. The better fitting random-effects-only model was then compared against the same model with the blur grade (polynomial, fitted as poly (blur_grade,2)) as a fixed effect. In the case of dyad/participant not converging or showing singular model fit, this was changed to a global participant number, rather than the nested dyad/participant. Models are reported as significant when they are a better fit to the data than the null model, as indicated by chi-square model comparison. Models containing the polynomial are additionally tested to ensure they are a better fit than the same model containing only the linear term.

We additionally used the MuMIn [60] package in *R* (functions *squaredGLMM* and *squaredLR*) to calculate pseudo-*R*^2^ values for any significant models. These values provide an estimate of the explained variance of the model. We report three values, which are informative in different ways: *R*^2^C (conditional *R*^2^) is the variance explained by the whole model, including fixed and random effects. We report this value for the full model of interest, and we additionally calculate a Δ*R*^2^ value, which is the *R*^2^C of the full model minus the *R*^2^C of the null model, providing an indication of how much variance is explained by the inclusion of visual quality as a fixed effect. As an alternative measure of this improvement of model fit, we calculate *R*^2^LR, which calculates the *R*^2^ based on a likelihood ratio test between the full model and null model.

For *motion energy* analyses, we tested the effect of visual quality on motion energy in two steps. The first analyses tested for an overall relationship between visual quality and motion energy in general, across all articulators. If this was significant, we added an interaction term between visual quality and articulator (four levels: torso, head, arms and fingers). If this was a significantly better fit than without articulator (see statistical testing explanation below), we proceeded to test the individual articulators separately in our second set of analyses. See above for more details on statistical modelling.

For *speech-gesture alignment* analyses, coherence and phase asynchrony values were entered into polynomial mixed effects regression analyses, as described above for the motion energy analyses, allowing us to determine whether the strength of coupling (i.e. Coherency) or the temporal nature of the coupling (i.e. Phase Asynchrony) is influenced by visual quality. See electronic supplementary material for a more detailed description of these methods.

*Gesture kinematics* were tested according to the general description given above. However, given that the kinematic features are characterized by different distributions (e.g. count data for submovements, accumulated time for hold-time), we used generalized linear mixed models whenever this led to a better model fit, as determined by examining the model residuals [61]. Following this procedure, we used generalized linear mixed models with a Poisson distribution for submovements, and with a log gamma distribution for peak velocity, maximum distance, size, depth, temporal variability and hold-time. Gesture-only versus gesture-with-speech was not included in the models, as we did not expect many cases of gesture-only occurrences. We implemented cumulative-link mixed models (clmm) from the statistical package ‘Ordinal’ [62] to test McNeillian Space, due to the ordinal nature of the data. Cumulative-link models were built up and tested in the same way as the other signal-level tests. The only difference being that likelihood ratio statistics and *z*-values are given for this test rather than chi-square and *t*-values.

For *speech acoustics*, we first tested a model with a dichotomous speech-only versus speech-with-gesture value as the independent variable, before testing for an effect of blur grade on F0 and intensity. This was done to determine whether speech acoustics were influenced by the co-presence of gesture. If this model showed a significant fit to the data, we then tested whether including blur grade further improved the model. This procedure ensured that any effect of visual quality on speech acoustics could not be explained by whether or not a gesture co-occurred with the speech. However, as the effect of modality is interesting in itself, we included an additional step, if the visual quality model was significant. In this case, we tested our visual quality model with a model additionally including an interaction between visual quality and modality. This allowed us to test not only whether the effect of visual quality is over and above any differences due to modality, but also whether visual quality has a modality-specific effect on speech acoustics. As a final step, in the case of a significant effect of blur grade on speech acoustics, we additionally compared a model with only blur grade as an independent variable against a model with both blur grade and a kinetic predictor. The kinetic predictors that we used are gesture peak velocity, as well as motion energy of the head, hands and torso (in separate models). This was to determine whether any changes in speech acoustics could be explained purely (or largely) by an account of speech acoustic changes being driven by the biomechanical coupling of the vocal system with postural or stabilizing muscles. In this account, peak forces in a gesture movement lead to stabilizing muscle activity throughout the torso, compressing the lungs and thus leading to co-occurring peaks in speech intensity and pitch [35]. If we find evidence for the model including gesture velocity or motion energy as being a strong predictor of speech intensity, this would suggest that speech adaptation may actually be due to this biomechanical coupling, rather than being a true adaptation to the communicative environment.

Tests were considered significant when below an alpha level that was adjusted for multiple comparisons within each family of tests. The corrected alpha was calculated using the online Simple Interactive Statistical Analysis tool (https://www.quantitativeskills.com/sisa/calculations/bonfer.htm) which takes into account both the number of tests and the average correlation between values to calculate a new Bonferroni-corrected alpha level. The four motion energy features showed an average correlation of 0.342, leading to an adjusted alpha of 0.024. The two speech acoustic features showed an average correlation of 0.002, leading to an adjusted alpha of 0.025. The six gesture kinematics features showed an average correlation of 0.283, leading to an adjusted alpha of 0.014.

## 3. Results

### 3.1. Articulator level

**Figure 3.**
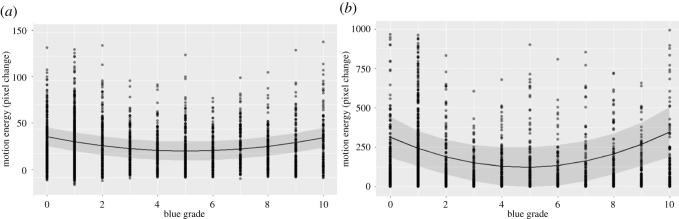
Quadratic relationships between visual quality and motion energy. In both panels, blur grade is given on the *x*-axis, and motion energy on the *y*-axis; (*a*) depicts motion energy of the arms. *Y*-axis limit is set to 150 for readability (not showing 0.8% of data points); (*b*) depicts motion energy of the torso. *Y*-axis limit is set to 1000 for readability (not showing 2% of data points). In both panels, the solid lines give the regression curve, while the shaded area gives the 95% confidence interval. Circles depict individual data points.

Our first analysis tested whether there was an association between visual quality and motion energy overall, and whether there was evidence for the effect of visual quality differing across the different articulators (i.e. an interaction effect). For these analyses, a single participant ID (40 levels) was used as a random effect, rather than the nested dyad/participant, due to a singular fit with the nested term. Our first model comparison showed a quadratic relationship between visual quality and motion energy, $x^{2}(2)=14.711$, *p* < 0.001. Our second model comparison added an interaction between visual quality and articulator (head, torso, arms and fingers). This effect was also significant, $x^{2}(5)=15.380$, *p* = 0.009. In terms of explained variance, the final model had an *R*^2^C = 0.043, *R*^2^LR = 0.017 and Δ*R^2^C* = 0.017. See figure 3 for an overview of the significant results. Note that the *y*-axis is limited in these plots for readability, but the model results did not change when the same outlying data points were removed (results not shown here). Our second analysis investigated the individual articulators, modelling each articulator's motion energy as the dependent variable in separate models. We found a significant quadratic effect of visual quality on torso movement, $x^{2}(2)=10.899$, *p* = 0.004. This model was further improved by including random slopes between participant ID and visual quality, $x^{2}(5)=480.82$, *p <* 0.001. The final model showed *R*^2^C = 0.859, *R*^2^LR = 0.128 and Δ*R^2^C* = 0.773, as well on arm movement, $x^{2}(2)=47.167$, *p* < 0.001. Including random slopes between dyad/participant and visual quality further improved model fit, $x^{2}(10)=156.52$, *p* < 0.001. The final model showed *R*^2^C = 0.683, *R*^2^LR = 0.055 and Δ*R^2^C* = 0.502. In both cases, there was a U-shaped relationship between the two variables (figure 3), meaning that motion energy first decreased as the blurring set in and then increased again during the stronger blur grades. We additionally performed a post hoc analysis with motion energy as the dependent variable and articulator (torso versus arms) as primary independent variable, with an interaction between articulator and blur grade. This was done to assess whether the shape of the response differed between the two articulators. This model showed a significant fit to the data, $x^{2}(3)=80.989$, *p* < 0.001. This can be seen in figure 3 as the relatively stronger quadratic effect of visual quality on torso compared to arms (*β* = 3814 ± 1391 pixels, *t* = 2.741). We found no effect of visual quality on head movement, $x^{2}(2)=3.147$, *p* = 0.207, or on finger movement, $x^{2}(2)=3.875$, *p* = 0.144.

In sum, the analyses on the articulator level showed a significant effect of visual quality on the amount of both arm and torso movement, with movement in both articulators first decreasing as visibility decreases, and then increasing again.

### 3.2. System level

We found no significant evidence for a positive linear effect of visual quality on speech-gesture **coherence** (i.e. no stronger coherence with increasing blur), $x^{2}(5)=9.853$, *p =* 0.095, accounting for random slopes gesture events, and no evidence for a quadratic relationship, $x^{2}(7)=7.219$, *p* = 0.406.

We found no significant evidence for a linear relationship between visual quality and speech-gesture **phase asynchrony** (i.e. greater phase asynchrony with increasing blur)**,** $x^{2}(3)=6.390$, *p* = 0.094, accounting for random slopes across gesture events, and no evidence for a quadratic relationship, $x^{2}(7)=4.552$, *p* = 0.103.

Thus, visual quality does not appear to impact on temporal gesture-speech coordination at the system level.

### 3.3. Signal level: speech

**Figure 4.**
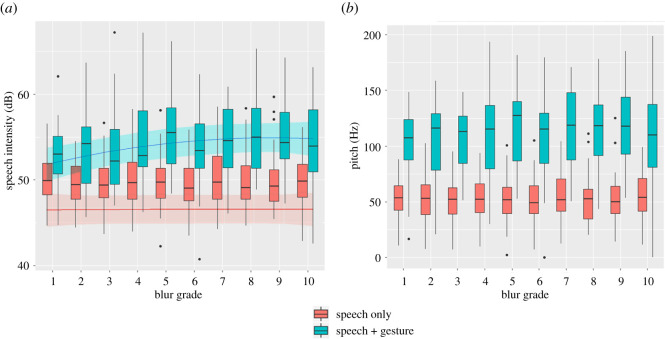
Speech acoustics across blur grades, split per modality; (*a*) represents speech intensity, while (*b*) represents pitch. In both plots, blur grade is given across the x-axis, and red bars depict speech-only utterances, while blue bars depict speech-gesture utterances. Boxes span the first to third quartile of the data, with the middle bar providing the median, and the whiskers showing the minimum and maximum of the data. Circles depict outlying data points; (*a*) additionally shows the fitted regression curves (solid line) within a 95% confidence interval (shaded area).

For speech, we found a main effect of modality (speech-gesture versus speech-only) on speech **intensity**, $x^{2}(1)=325.02$, *p* < 0.001, with intensity being higher for speech-gesture than speech-only utterances. Intensity was further affected by visual quality $x^{2}(2)=8.2637$, *p* = 0.016, with speech intensity increasing as visual quality decreased. By adding an interaction between visual quality and modality, the model again improved in fit, $x^{2}(2)=7.888$, *p =* 0.019. In this final model, we see that intensity increased by 4.36 ± 0.2 dB in speech-gesture compared to speech-only utterances (*t* = 20.49). The change in intensity across blur grades revealed a quadratic increase, which was primarily driven by speech-gesture utterances, which showed that as visual quality decreased, the increase in speech-gesture utterances was 15.6 ± 6.1 dB greater than the visual quality-related increase in speech-only utterances (linear: *t* = 2.57; quadratic: *t* = −1.06). The final model had an *R*^2^C = 0.551, *R*^2^LR = 0.359 and Δ*R*^2^*C* = 0.259. See figure 4*a* for a graphical representation of this relationship. **Pitch** was similarly affected by modality, $x^{2}(1)=25.572$, *p* < 0.001, with pitch increasing by 3.71 ± 0.73 Hz in speech-gesture compared to speech-only utterances (figure 4*b*). We found no evidence for pitch being further modulated by visual quality, $x^{2}(2)=2.827$, *p* = 0.243.

Finally, to determine whether speech intensity was being driven by co-occurring gesture kinetic forces, we compared a model including only a quadratic effect of visual quality on speech intensity against a model including both this quadratic effect and kinetic predictors (peak velocity and motion energy). We found no evidence for gesture peak velocity significantly contributing to the changes in speech intensity, $x^{2}(1)=0.391$, *p* = 0.532. We additionally tested the same null model against models containing head ($x^{2}(1)=1.359$, *p* = 0.244), hand ($x^{2}(1)=1.066$, *p* = 0.302) and torso ($x^{2}(1)=0.139$, *p* = 0.708) motion energy, and similarly found no significant effects. These results suggest that the quadratic intensity increase was driven by the decrease in visibility directly rather than by changes in gesture or bodily movement, which could have affected speech parameters.

In sum, speech intensity is affected by visual quality, but only when gestures are present, and it does not seem to be driven by the kinetic forces of gesture or bodily movement per se. Pitch is affected by the presence of gesture, but not by visual quality.

### 3.4. Signal level: gesture

**Figure 5.**
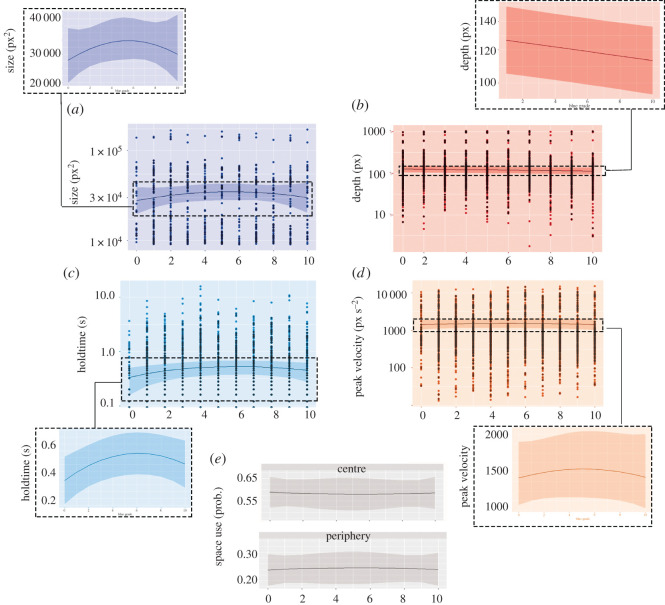
Relationship between blur grade and gesture kinematics; (*a*) depicts size, (*b*) depicts depth, (*c*) depicts hold-time, (*d*) depicts peak velocity, and (*e*) depicts McNeillian Space. In all graphs, blur grade is given along the *x*-axis. In (*a*,*b*,*c* and *d*), the kinematic value is given on the *y*-axis (in log10 scale), while in (*e*) the *y*-axis depicts the probability of a gesture occurring in the given space value; (*e*) depicts the centre and periphery spaces as these accounted for most of the probability distribution. In all panels, the solid lines give the regression curve, while the shaded area gives the 95% confidence interval. Circles depict individual data points. For (*a*,*b*,*c* and *d*), there is additionally a cut-out providing a zoomed-in visualization of the predicted regression curve. Note that no data points are shown for (*e*), given that values on the *y*-axis are based on probability, rather than actual data points.

Gesture rate was significantly modulated by visual quality, $x^{2}(2)=24.83$, *p* < 0.001, with an increase in overall **gesture rate** through the first five blur grades, followed by a decrease in the last five blur grades (positive quadratic effect). Our tests of kinematics revealed that visual quality showed a positive quadratic relationship with **peak velocity**, $x^{2}(7)=118.75$, *p* < 0.001 (figure 5*d*), as well as with **hold-time**, $x^{2}(2)=9.497$, *p* = 0.009 (figure 5*c*), and **size**, $x^{2}(7)=37.871$, *p* < 0.001 (figure 5*a*). In these cases, kinematic values increased during the initial five blur grades before decreasing again across the last five blur grades. **McNeillian Space** similarly showed a quadratic relationship with visual quality, likelihood ratio (12) = 82.97, *p* < 0.001; probability of peripheral space use increased initially before decreasing, while the probability of central space use first decreased before increasing, but note the small effect size—figure 5*e*. Visual quality showed a negative linear relationship with **depth,** $x^{2}(2)=9.405$, *p* = 0.009 (figure 5*b*). We found no effect of **temporal variability**, $x^{2}(2)=0.392$, *p* = 0.822, and no effect of visual quality on **submovements**, $x^{2}(1)=2.129$, *p* = 0.145. A summary of the statistically significant final models and the associated fixed-effect coefficients and statistics can be found in table 1, and the significant findings can be seen in figure 5.

**Table 1.** Overview of model outcomes for significant kinematic features. *R*^2^C = conditional *R*^2^ (based on full model); Δ*R*^2^C = conditional *R*^2^ of the full model minus conditional *R*^2^ of the null model; *R*^2^LR = likelihood ratio *R*^2^, based on improvement of the full model from the null model.

kinematic feature and final model	term	*β* ± std. error	*T*	*R^2^C*	Δ*R^2^C (compared to null model)*	*R^2^LR*
peak velocity velocity ∼ poly(blur_grade,2) + (1 + poly(blur_grade,2)|subject) + (1|gesture_type), family = gamma(link = log)	linear	−0.019 ± 1.95	−0.010	0.14	0.0218	0.034
	quadratic	−1.454 ± 1.92	−0.757			
hold-time hold-time ∼ poly(blur_grade,2) + (1|dyad/participant) + (1|gesture_type)	linear	1.890 ± 1.0	1.854	0.0744	0.0023	0.003
	quadratic	−2.500 ± 1.0	−2.460			
size size ∼ poly(blur_grade,2) + (1 + poly(blur_grade,2)|subject) + (1|gesture_type), family = gamma(link = log)	linear	−0.592 ± 1.922	0.308	0.2615	0.1455	0.053
	quadratic	−1.551 ± 1.562	−0.993			
depth	linear	−0.01 ± .004	−2.984	0.1829	0.009	0.003
depth ∼ poly(blur_grade,2) + (1|subject) + (1|gesture_type), family = gamma(link = log)						
	term	*β* ± std. error	*Z*			
McNeillian space MN_mode ∼ poly(phase,2) + (1 + poly(phase,2) |dyad/participant)	linear	0.213 ± 3.5	0.061			
	quadratic	−0.833 ± 4.5	0.851			

Together, the signal-level analyses thus showed that, in addition to gesture rate, decreasing visual quality significantly affected gesture kinematics in terms of peak velocity, hold-time, size and depth (and to some extent McNeillian space).

## 4. Discussion

The aim of this study was to quantify how visual quality affects the way that we use communicative signals. Specifically, we assessed whether speech also supports gesture, or only vice-versa, and whether gesture adapts to environmental challenges that are not associated with compromised verbal communication. We further assessed how speech and gesture dynamically interact during this process of adaptation. Finally, we assessed whether any speech-gesture adaptations scale with the degree of visual disruption.

To capture the multi-scale nature of communicative adaptation, we assessed the speech-gesture system at three levels of granularity: articulator level (i.e. distribution of movement across the torso, arms, hands and fingers), system level (i.e. the coupling of speech and gesture) and signal level (i.e. acoustics and kinematics). We found that there is a distinct pattern of kinematic and acoustic adaptation, with strong effects of speech intensity and gesture size increasing in the first five blur grades before decreasing again in the last five. At the same time, motion energy of the arms and torso showed the opposite pattern, decreasing at first before increasing again in the last five blur grades. Analyses on the system level showed the temporal coupling of speech and gesture seems not to be affected by visual quality.

### 4.1. Multi-modal adaptation as a two-way street

We found that speech acoustics are primarily adapted to visual quality when gestures are present. In other words, when decreased visibility is not an issue due to there being no gestures, speech is not adapted. However, when there *are* co-speech gestures, speech acoustics increase as visibility decreases. This increase in speech acoustics seems to primarily occur in the first grades of visual blurring, after which it plateaus. This finding indicates that speech acoustics are only adapted until the point that vision is very obscured, when gestures are also unlikely to be communicatively effective. Although these results may appear to be a simple effect of gesture per se leading to increased speech intensity, results from our interaction model suggest that this effect cannot be explained by the presence of gesture alone. Instead, it is the interaction between gesture and decreasing visual quality. Furthermore, we found no evidence for speech intensity being associated with gesture kinematics. This means that a decrease in visual quality is associated with an increase in speech intensity only when gestures are communicatively effective, and also not simply due to biomechanical coupling between speech and gesture [35]. This corroborates the idea that speech is also used to compensate for degraded gesture visibility, just as gesture can compensate for a degraded auditory signal.

### 4.2. Multi-scale adaptation

Regarding the general pattern of adaptation, an all-or-nothing type response to the general communicative context (e.g. compromised visibility resulting in a strong reduction in gesture versus good visibility resulting in gesture being used) would indicate that communicative signals are adapted in a more coarse-grained and less controlled manner. In this case, we may expect a linear increase in signal features as visual quality decreased. On the other hand, a graded adaptation would indicate that signal cost may be balanced at the level of these signal features and scaled to fit the specific communicative context. In this case, we would expect a nonlinear response in which signal features are gradually adapted to decreasing visual quality up to a point where adaptation is no longer likely to help, after which adaptation flattens out or returns towards baseline levels. We found the latter to be the case.

At the **articulator level**, we see that both torso movement and arm movement have a U-shaped quadratic association with visual quality. In other words, motion energy decreases together with visual quality until around the fifth (moderate) blur grade, before inflecting and increasing again across the highest, most severe blur grades. The finding of decreased motion energy builds on the signal-level results (discussed below) in that it shows that even when gestures overall occupy more space, there is less actual movement within this space. This suggests that speakers may adapt gesture kinematics to be more salient in terms of spatial prominence and temporal structure, but minimize the amount of movement involved. In other words, rather than producing gestures with many smaller movements, speakers use gestures with a few large movements. Our motion energy findings also show that people reduce the movement of not only their arms, but also their torso. This highlights how although the gesturing hands are one of the most commonly studied aspects of multi-modal communication, the dynamics that we observe as a response to communicative demand extend across multiple scales and seem to involve the entire body. These findings further underline the interconnectedness and multi-scale nature of multi-modal communication [63,64].

At the **system level** of speech-gesture alignment, we found no evidence for visual quality affecting how strongly speech and gesture are coupled, or in the temporal relationship between the two. Although one previous study has shown visibility to affect speech-gesture coupling [36], it should be noted that this study differed from ours in at least two substantial ways. First, Wagner and Bryhadyr used a much more task-focused approach and analysed specific speech-gesture, or more specifically speech-*action*, events. Namely, the authors looked at the moment a game piece was being moved and the description of this move was being uttered. The second major difference is that the Wagner and Bryhadyr study used only two conditions, visible and occluded. Both of these differences could have contributed to the lack of findings in the present study, as speech-gesture coupling effects may be specific to key utterances that the speaker is emphasizing. Similarly, coupling effects may be specific to when one knows they are completely occluded, compared to when there is a visual channel open to the addressee, but the quality of the channel is poor. Taken together, it seems that the mutual visibility has a nuanced effect on speech-gesture coupling that, as we see in the current study, may not generalize to speech-gesture utterances occurring in natural, unstructured conversation.

At the **signal level**, we found that both speech acoustics and gesture kinematics were affected by visual quality. In the speech acoustics, we found that intensity increased as a function of decreasing visual quality, while pitch did not seem to depend on visual quality. This fits with the findings of intensity acting as a relatively general adaptation to adverse communicative situations [2,65], while pitch may not always be adapted. Instead, pitch adaptation is more specific to auditory noise, where increasing pitch can actually be beneficial [66]. As discussed above, speech intensity also showed a quadratic relationship with visual quality, when paired with gestures. Interestingly, this suggests that the speech acoustic modulation, which seems to support the visual channel, is adapted to the specific level of visual quality. Furthermore, follow-up analyses indicate that although speech intensity is greater when gestures are present, the speech intensity adaption cannot be explained by the general bioacoustic coupling between speech and gesture. This suggests that speech intensity, similar to gesture kinematics (see below), is influenced by mutual visibility when the visual channel is particularly relevant (i.e. when manual gestures are being performed). Note that these results do not necessarily contradict earlier findings that may indicate a more linear relationship between speech intensity and environmental factors (e.g. auditory noise). Instead, our results primarily point to communicative context and ‘utterance context’ (i.e. whether speech is embedded in a more multi-modal context) together influencing the production of speech acoustics.

In the gesture kinematics, we found that decreasing visual quality was associated with larger, more temporally segmented gestures, at least in the moderately blurred condition. This can be seen in the gesture size, a measure of the overall three-dimensional space in which the hands operate in a given gesture, as well as the McNeillian space, which categorizes where in relation to the speaker's body the gesture took place. These measures showed that in the moderate levels of visibility, gestures became larger and were more likely to occur in the gestural ‘periphery’, away from the central area directly in front of the chest. These kinematic features then returned towards their baseline as blurring increased to the most severe conditions. We additionally found a reduction in gesture depth as visual quality decreased, as well as an increase in peak velocity and hold-time. However, despite the models themselves being significant, the effect of visual quality on these features was quite small, as exemplified by the low explained variance of these modes. Importantly, these changes occur primarily in the first five blur grades, but fall back off as blurring becomes severe. This suggests that these changes are adaptations to the visual context in which they were produced and are not used when the gestures are unlikely to be visible to the addressee.

These findings are in line with previous studies showing that gesture kinematics are adapted to the current communicative context [15,23,30,40,41,67–72]. Our study builds on these previous findings by showing that when the social context involves impoverished visibility, the adaptation follows a nonlinear, quadratic pattern, suggesting that the kinematic features are gradually adapted to the current communicative context, but only to the extent that the visual channel is still communicatively effective. Gestural kinematic adaption in response to a decrease in visibility thus appears to be a highly controlled process, resulting in a gestural response that is finely tuned to the specific environmental conditions.

While the present study assesses multi-modal communicative behaviour at multiple levels, future research should also consider interpersonal patterns of behaviour, such as interpersonal synchrony or mimicry [34,73,74]. Such high-level patterns may further influence effects at the level of communicative signals, motion energy and speech-gesture synchrony since they may vary depending on visibility. However, they are unlikely to provide an exhaustive explanation, since interactional synchrony, for example, occurs also in the absence of visibility [75].

### 4.3. General conclusions

This study has shown that, in unscripted, casual video-mediated conversation, when the visual channel is disrupted, the vocal channel is adapted to compensate. Our results therefore provide support for the notion that gesture is more than just a compensatory or supporting signal, but a core aspect of communication in its own right [25]. Besides showing that speech compensates for a disrupted visual channel, we demonstrate the balancing of spatio-temporal features of gesture kinematics. Specifically, spatial prominence and temporal structure are enhanced when visibility of gesture is reduced, while overall movement is decreased. We also provide evidence for the flexible specificity of this adaptation, as these effects primarily occur in the first five grades of visual quality, when such adaptation is likely to still benefit comprehension. As visual quality continues to decrease, gestural signals are no longer enhanced. This builds on previous evidence that gestures are flexibly and (partially) independently [15,76] adapted according to current communicative demands [41,77].

The present study is the first to investigate how visual quality affects natural communicative behaviour. Besides addressing the hitherto neglected research question of whether speech also compensates for a disrupted visual channel, we used naturalistic, unscripted conversation, thus enhancing the ecological validity of the findings. Furthermore, we tested the effect of visual quality across 10 grades of visual quality, thus increasing the resolution at which we were able to observe these effects and allowing us to assess the overall pattern of adaptation (i.e. all-or-nothing compared to controlled and graded). Finally, previous studies have shown that human communication is shaped by efficiency. In other words, there is a pressure for communicative systems (e.g. language) to achieve communicative success at the lowest possible signal cost [64]. This has been demonstrated in language features, such as the fact that more frequently used words tend to be shorter than less common words [78]. The dynamic of kinematic features being adapted to visual quality primarily when the gestures are still likely to be visible, with less adaptation as blurring becomes more severe, provides a first suggestion that gesture adaptation may also follow such an efficiency-based principle. The parallel adaptation of speech acoustics may be an interesting component of this behaviour, as increased speech intensity in response to reduced visibility is not in itself efficient or (perhaps) even useful. However, it may be a way to compensate for a weakening of the visual signal. Whether or not this behaviour is truly efficient requires future research that can measure communicative success (e.g. using problem-solving or referential tasks).

As an alternative to the efficiency-based interpretation discussed above, there may be an intermediate factor that influences both speech and gesture in the context of reduced visual quality. For example, it may be that certain parts of speech may be perceived as particularly relevant to convey, leading speakers to invest more communicative effort into those parts. This increased communicative effort could be expressed as increased speech intensity together with a higher chance of gesture production. In this case, the two modalities are somewhat independent in the sense that the kinetic forces of the gesture are not causing a change in speech intensity, but rather they are parallel responses to communicative demand. This explanation still allows for a nonlinear response of the two modalities, as this response can be seen as a general communicative response to how informative the visual channel can be, given the current visual quality.

While this study provides a unique, multi-scale investigation of communicative behaviour, the unconstrained, naturalistic paradigm also means that we can only find associations, and not causal links. Future studies should use more experimentally controlled designs in order to replicate and confirm these findings.

In sum, our results reveal how speech and gesture are core aspects of communication that are dynamically adapted to current communicative demands. In terms of the composition of the speech-gesture system, speech-gesture adaptation is a two-way street, with speech acoustics acting to compensate for disruption of the visual channel. Acoustic and kinematic adaptations also extend beyond signal level modulations, showing an involvement of the entire body. This study therefore provides new insights into how adaptation of the communicative system is a multi-scale, dynamically specified phenomenon.

## Data Availability

The analysis scripts and processed data are available at the following OSF project: https://osf.io/d8juc/?view_only=998f9522e12b4943b457498261e07aae.
